# Reliability, validity and dimensionality of the 12-Item General Health Questionnaire among South African healthcare workers

**DOI:** 10.4102/ajopa.v6i0.144

**Published:** 2024-10-17

**Authors:** Clement N. Kufe, Colleen Bernstein, Kerry Wilson

**Affiliations:** 1 Department of Epidemiology and Surveillance, National Institute for Occupational Health, National Health Laboratory Service, Johannesburg, South Africa; 2 School of Health Systems and Public Health, Faculty of Health Sciences, University of Pretoria, Pretoria, South Africa; 3 Department of Psychology, School of Human and Community Development, University of the Witwatersrand, Johannesburg, South Africa; 4 School of Public Health, Faculty of Health Sciences, University of the Witwatersrand, Johannesburg, South Africa

**Keywords:** healthcare workers, reliability, validity, 12-Item General Health Questionnaire, dimensionality

## Abstract

Healthcare workers (HCWs) were among the high-risk groups for severe acute respiratory syndrome coronavirus 2 (SARS-CoV-2) infection and suffer a high burden of challenges with mental health including depression, anxiety, traumatic stress, avoidance and burnout. The 12-Item General Health Questionnaire (GHQ-12) has shown the best fit in both a one-factor structure and a multidimensional structure for the screening of common mental disorders and psychiatric well-being. The aim was to test for reliability and validity and ascertain the factor structure of the GHQ-12 in a South African HCW population. Data were collected from 832 public hospital and clinic staff during the coronavirus disease 2019 (COVID-19) pandemic in Gauteng, South Africa. The factor structure of the GHQ-12 in this professional population was examined by exploratory factor analysis (EFA) to identify factors, confirmatory factor analysis (CFA) for construct validity and structural equation modelling (SEM) to establish model fit. The GHQ-12 median score was higher (*n* = 25) in women than in men (*n* = 24), *p* = 0.044. The assumptions for inferential statistics were tested: the determinant for the correlation matrix was 0.034, Bartlett’s test of sphericity was *p* < 0.001, Chi-square 2262.171 and the Kaiser-Meyer-Olkin (KMO) of sampling adequacy was 0.877. The four factors identified were labelled as social dysfunction (37.8%), anxiety depression (35.4%), capable (24.9%) and self-efficacy (22.7%). The sample had a Cronbach’s alpha and McDonald’s Omega coefficient of 0.85.

**Contribution:**

The study highlighted the gaps in the use of GHQ-12. The findings affirm the validity and reliability of the GHQ-12 in this group of professionals and the multidimensional structure for screening for psychological distress.

## Introduction

The coronavirus disease 2019 (COVID-19) arose in China in December 2019 and by early February 2020 reports of psychological pressures on Chinese healthcare workers (HCWs) were published (Kang et al., 2020). The coronavirus disease 2019 did not only affect the mental health of Chinese HCWs but also their physical health as it rapidly spread throughout the world. By the 11th of March, the World Health Organization (WHO) had declared COVID-19 a global pandemic (Shaukat et al., 2020). The first recognised COVID-19 case was diagnosed in South Africa on 05 March 2020 and 116 cases were registered by 18 March 2020. Following the National State of Disaster declared on the 15th of March, with schools closing on the 18 March 2020, a countrywide lockdown began on the 26 March 2020 in South Africa. South African HCWs were designated essential services and continued working at the forefront in the fight against the COVID-19 pandemic. Healthcare workers were responsible for screening, testing and managing COVID-19-positive patients as well as unknown COVID-19 patients, along with those responsible for cleaning healthcare facilities and, dealing with the administration and management of facilities (Lee et al., 2022). A high degree (57.4%) of psychological distress and a strong association between perceived risks associated with the presence of COVID-19 in the healthcare workplace and associated psychological distress was reported among HCWs in South Africa throughout the pandemic (Lee et al., 2022).

Epidemic outbreaks have been known to place an unprecedented demand on HCWs resulting in increased deaths, infection, shortage of medication and vaccines, increased workload and a lack of personal protective equipment, and feelings of inadequate support all exacerbating the mental distress and burden of HCWs (Chen et al., 2020; Lai et al., 2020; Lee et al., 2022). A systematic review of the mental health of HCWs during the COVID-19 pandemic reported the lowest prevalence of anxiety, depression and stress as 24.1%, 12.1% and 29.8% in HCWs from Fujian province in China and the highest prevalence of 67.6%, 55.9% and 62.9% in Spain for cross-sectional studies published in English (Vizheh et al., 2020). Another review of studies at the time identified risk factors for COVID-19-related health impact in HCWs being: inadequate hand hygiene, high-risk department, diagnosed family member, suboptimal hand hygiene before and after contact with patients, improper personal protective equipment use, close contact with patients (≥ 12 times/day), long daily contact hours (≥ 15 h) and female HCWs disproportionated affected (Shaukat et al., 2020).

Because of stigma, HCWs were likely to experience psychological distress in silence, which may have led to an increased risk of suicidal ideation (Dutheil et al., 2019; Knaak et al., 2017; Mehta et al., 2018). The uneasy climate during COVID-19 characterised by panic and fear in the general population was likely increased in HCWs as they needed to manage patient care at a time when their own physical and mental well-being was at also stake (WHO, 2020). The unremitting stress of the pandemic or epidemic could have led HCWs to experience elevated levels of anxiety, fear, panic attacks, posttraumatic stress symptoms, psychological distress, stigma, avoidance of contact, depressive symptoms, sleep disturbances, feelings of helplessness, interpersonal isolation from family social support, and concern regarding contagion exposure to their friends and family (Jawad et al., 2021; Rana et al., 2020; Søvold et al., 2021). At the same time, some programmes put in place faced resistance from HCWs in acknowledging the experience of psychological difficulties (Buselli et al., 2021). Therefore, a need to provide evidence-informed data on the mental health and well-being effects of HCWs to mitigate these challenges and provide evidence-informed recommendations for the future as the world transitions out of the pandemic (Søvold et al., 2021).

The General Health Questionnaire (GHQ) developed in the 1970s was initially intended for use as a unidimensional tool of 60-items to describe the risk of mental disorders. Many shortened versions of the GHQ were developed such as the GHQ-30, the GHQ-28, the GHQ-20 and the GHQ-12 (Burvill & Knuiman, 1983; Goldberg, 1972, 1989; Illness et al., 1972), all of which have been subject to factor analytic procedures to identify whether each of the scaled versions provide additional utility (Kline, 2014). The shorter GHQ-12 was used in epidemiologic studies, the majority of which and subsequent factor analytic studies failed to accept the unitary construct (Corti, 1994) and proposed a multidimensional structure (Werneke et al., 2000).

Because of its brevity, the 12-item GHQ has been widely used as a screening instrument for the presence of mental disorders in many countries (Donath, 2001; Doyle et al., 2012; Hu et al., 2007; Illness et al., 1972; Lundin et al., 2016; Özdemir & Rezaki, 2007; Uras et al., 2012). While the GHQ has been widely used to date, no study could be identified that had examined the factor structure on a South African sample.

The factor analytic approach assesses interrelationships within a set of variables to construct a smaller number of hypothetical variables or factors that contain the essential information of the larger set of observed variables, thus reducing the overall complexity of the data by taking advantage of the inherent interdependencies (Campbell et al., 2003). Factor analysis is useful for measuring constructs that are not readily observable, summarising large observations into smaller numbers and providing evidence of construct validity by hypothesis testing (Kline, 2014). Studies that have been carried out to assess the mental health of HCWs including the use of the 12-Item General Health Questionnaire (GHQ-12) have not been validated in all professional populations. This study subsequently aimed to assess the validity and reliability and determine the factor structure of the GHQ-12 in the South African, Gauteng, healthcare worker population.

## Methods

### Study design

A cross-sectional study was conducted in three public tertiary hospitals, two regional hospitals, two community health centres and nine clinics in Gauteng, South Africa between August and October 2020.

### Participants

Data were collected from a convenience sample of 832 male and female hospital staff during the COVID-19 pandemic. The median (25th–75th percentile) age of the respondents was 44 (34–54) years, 81.7% identified as female, 17.9% as male and 0.4% as another. Furthermore, 99% were South African citizens and the remainder were from Southern Africa. The most common home language was Venda, followed by Setswana and IsiZulu. Majority (33.6%) of the participants were nurses, 11% were medical doctors, 8.8% were radiologist, 2.4% were either physiotherapists or pharmacists, 2.6% were clerks, 10.4% were working in the delivery department and the remaining 30.2% were involved in a variety of functions.

### Instruments

A self-administered GHQ-12 hard copy and electronic questionnaire exploring socio-demographic information, assessing stress and psychological effects, perceptions, attitudes and behaviour around COVID-19 was used. The GHQ-12 consisted of 12 statements to which respondents indicated agreement on a four-point scale (1 = Better than usual, 2 = Same as usual, 3 = Worse than usual and 4 = Much worse than usual) on mental stress (Goldberg, 1972).

### Procedure

The GHQ-12 and others were provided to the Gauteng Department of Health, Central Office, and Wellness Unit from where it was distributed to the Wellness Coordinators of the Johannesburg facilities. The questionnaire was printed and the Wellness coordinators distributed it to HCW from all the job categories and collected the completed questionnaires. The returned questionnaires were sent to the National Institute for Occupational Health (NIOH) for capturing and analysis. The online version of the questionnaire was available on Research Electronic Data Capture (REDCap), and the link was provided to the coordinators. Data were captured on REDCap (Harris et al., 2009).

### Data analysis

Data were analysed using Stata version 16.1/MP (StataCorp, College Station, TX, United States [US]) after exploration to establish missing data points. The univariate normality of the items was assessed using the skewness and kurtosis of the responses. To establish the interrelatedness of the items for internal consistency and adequacy, an acceptable Cronbach’s alpha and a McDonald Omega of at least 0.70 was adopted. McDonald’s omega is unbiased for congeneric items with uncorrelated errors unlike Cronbach’s alpha (Malkewitz et al., 2023; Padilla & Divers, 2016). To ascertain whether the data satisfied the assumptions for inferential analysis, the Bartlett’s test supplemented by the Kaiser-Meyer-Olkin (KMO) measure of sample adequacy was used for the factor analysis with significant results and a KMO > 0.50, respectively.

Both exploratory and confirmatory factor analyses were implemented. Factor analysis was used to examine the theory-driven factor structure as evidential criteria using exploratory factor analysis (EFA) (Gorsuch, 1983; Lloret et al., 2017; Watkins, 2018) and by testing the assumptions using confirmatory factor analysis (CFA) to establish model fit (Loehlin, 2017; Russell, 2002; Young & Pearce, 2013). The use of EFA permitted a compromise by balancing the parsimony and comprehensiveness in the model that contains just enough factors to explain the important variations in the measured variables and later using multiple methods to advise on the plausible and appropriate factor solutions (Fabrigar et al., 1999; Gorsuch, 1983; Hair et al., 2010; Henson & Roberts, 2006; Lloret et al., 2017; Loehlin, 2017; Norris & Lecavalier, 2010). Using EFA, the appropriate factors to explain the relationship between observed variables were established. Orthogonal (varimax) was employed to determine the number of factors to retain by using the following criteria: (1) eigenvalues of the factors ≥ 1, (2) Cattell’s scree test, (3) internal consistency and (4) factors that yielded meaningful psychological constructs. Structural equation modelling (SEM) assessed the predictive utility of the factors identified with CFA by using maximum likelihood estimation for the following goodness-of-fit criteria: Likelihood ratio, population error, information criteria, baseline comparison and size of residuals (Hooper et al., 2008).

### Assessment of model fit, comparison and predictive utility

Confirmatory factor analysis was used to ascertain whether the factor structure that was developed from the data matched the chosen conceptual models and an analysis goodness-of-fit test between the data and the model (Reymont & Jvreskog, 1993). Model fit was evaluated by examining the size and statistical significance of the factor loadings as well as the comparative fit index (CFI), Tucker-Lewis index (TLI), the standardised root mean square residual (SRMR) and the root mean square error of approximation (RMSEA) at 90% confidence interval (CI).

An RMSEA of 0.01, 0.05 and 0.08 is generally considered to represent excellent, good and mediocre fit, respectively, at 90% CI (MacCallum et al., 1996) while an SRMR of less than 0.08 is typically considered to be a very good fit while 0.05–1.0 was considered an acceptable fit (Hu & Bentler, 1999). A CFI and TLI close to 0.95 represents a good fit between the hypothesised model and the data while values in the range of 0.90–0.95 are acceptable (Hu & Bentler, 1999). Kline, 2015; McDonald & Ho, 2002). Competing models were compared by use of the Akaike’s information criterion (AIC), the Bayesian information criterion (BIC) and the likelihood ratio test where the lower values for AIC and BIC indicate a better fit in tandem with the parsimony goodness-of-fit index having the highest overall coefficient of determination (CD) or *R*-squared (Hooper et al., 2008).

### Ethical considerations

Ethical approval to conduct the study was obtained from the University of the Witwatersrand, Human Research Ethics Committee (Medical) Clearance (certificate no.: M2006103). A wellness coordinator in each health facility invited the HCWs who provided consent and were enrolled in the study. Written consent was obtained from all participants before completing the anonymous online questionnaire.

## Results

The GHQ-12 median score was 25 for females as compared to 24 for males and the difference was statistically significant, *p* = 0.044. The determinant for the correlation matrix was 0.034, the Bartlett’s test of sphericity was significant (*p* < 0.001) with a Chi-square of 2262.171 and the KMO of sampling adequacy of 0.877 confirming the suitability of data for the factor analytic technique. The entire sample had a general average inter-item covariance of 0.33 and a Cronbach’s alpha of 0.85, 0.85 for males and 0.84 for females illustrating satisfactory internal consistency in both groups and in males and females, respectively. For the McDonald’s omega coefficient, it was 0.85 for both males and females, 0.86 for males and 0.84 for females. The range of the item-scale correlation was 0.50 for ‘playing a useful part in things’ to 0.69 for the ‘unhappy’ and ‘depressed’ in the entire sample (Table 1). The values of Cronbach’s alpha of 0.85 and the McDonald’s omega of 0.85 were comparative or comparable. The majority of respondents completed all 12 questions, although ‘Been able to enjoy normal day-to-day activities’ was the most skipped question.

**TABLE 1:** Item-scale analysis of the 12-Item Global Health Questionnaire.

GHQ-items	*n*	AISC	α
1. Been able to *concentrate* on what you are doing?	847	0.59	0.84
2. Lost much *sleep* over *worry*?	836	0.61	0.84
3. Felt that you are playing a *useful* part in things?	830	0.50	0.85
4. Felt *capable* of making *decisions* about things?	831	0.57	0.84
5. Felt constantly under *strain*?	846	0.66	0.84
6. Felt that you could not overcome your *difficulties*?	838	0.68	0.83
7. Been able to *enjoy* your normal day-to-day *activities*?	829	0.61	0.84
8. Been able to *face* up to your *problems*?	844	0.61	0.84
9. Been feeling unhappy and *depressed*?	846	0.69	0.83
10. Been *losing confidence* in yourself?	850	0.68	0.83
11. Been thinking of yourself as a *worthless* person?	849	0.59	0.84
12. Been feeling reasonably *happy* all things considered?	832	0.62	0.84

AISC, adjusted item-scale correlations; GHQ, global health questionnaire.

α examines reliability by determining the internal consistency of a test or the average correlation of items (variables) within the test.

### Reliability, validity and the factor structure

For the factor analysis, 672 participants were included, 4 factors were retained, and all had a coefficient greater than 0.30. The test of internal consistency represented by a scale of reliability coefficient was 0.85 for all the 12 items, 0.78 for Factor I, 0.76 for Factor II, 0.64 for Factor III and 0.64 for Factor IV in orthogonal (varimax) rotation. The data satisfied the assumptions for inferential analysis in this population. The factor loadings were further labelled as follows: Factor I labelled ‘Social Dysfunction’ (37.8%); Factor II called ‘Anxiety and Depression’ (35.4%); Factor III called ‘Self-Efficacy’ (22.7%) and Factor IV labelled ‘Capable’ (24.9%) with the first three factors accumulatively representing 98.1% (Table 2).

**TABLE 2:** Maximum likelihood estimates for orthogonal (varimax) factor loadings of the 12-Item General Health Questionnaire.

GHQ-items	I	II	III	IV
1. Been able to *concentrate* on what you are doing?	0.31	-	-	0.39
2. Lost much *sleep* over *worry*?	0.45	-	-	0.30
3. Felt that you are playing a *useful* part in things?	-	-	-	0.49
4. Felt *capable* of making *decisions* about things?	-	-	-	0.50
5. Felt constantly under *strain*?	0.62	-	-	-
6. Felt that you could not overcome your *difficulties*?	0.55	-	-	-
7. Been able to *enjoy* your normal day-to-day *activities*?	0.42	-	0.45	-
8. Been able to *face* up to your *problems*?	-	-	0.49	-
9. Been feeling unhappy and *depressed*?	0.44	0.51	-	-
10. Been *losing confidence* in yourself?	-	0.66	-	-
11. Been thinking of yourself as a *worthless* person?	-	0.60	-	-
12. Been feeling reasonably *happy* all things considered?	-	0.34	0.41	-

GHQ, global health questionnaire.

Exploratory factor analysis using orthogonal (varimax) to determine the number of factors. The four-factor structure labelled as Factor I (Social Dysfunction), Factor II (Anxiety and Depression), Factor III (Self-Efficacy) and Factor IV (Capable).

**FIGURE 1:**
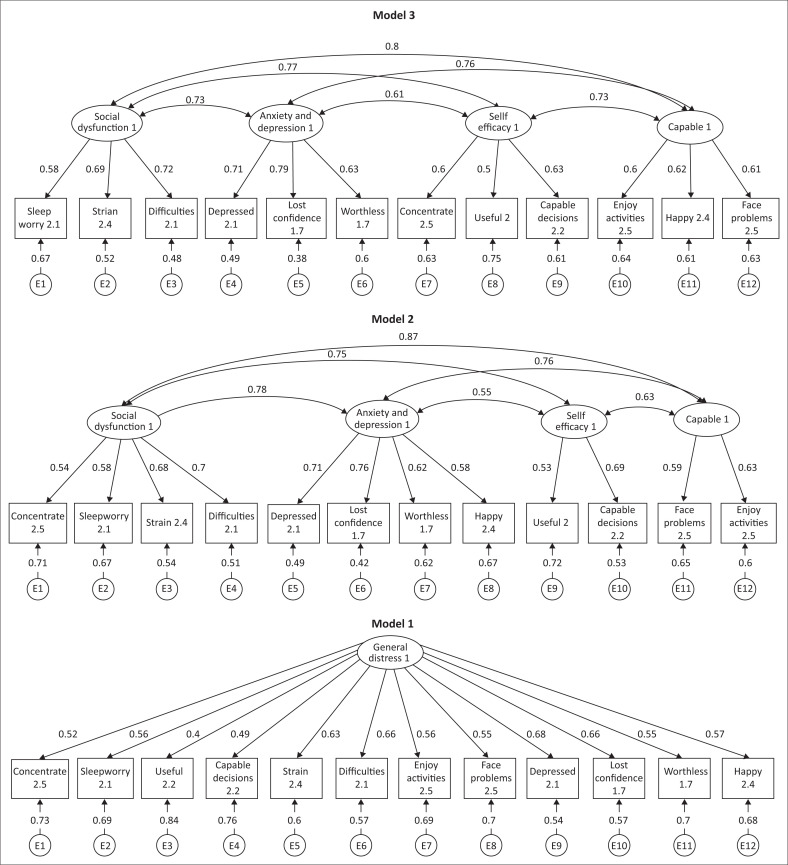
The different factor models for the 12-Item General Health Questionnaire compared in South African healthcare workers.

We examined the size and statistical significance of the factor loadings and common goodness-of-fit statistics. A unidimensional model was tested for all the 12 items in Model 1 named ‘Mental distress’. In Model 2, a four-factor structure was tested and labelled as ‘Social dysfunction’ (4 items), ‘Anxiety and Depression’ (4 items), ‘Self-Efficacy’ (2 items) and ‘Capable’ (2 items). While in Model 3, the four-factor structure was maintained but item distribution was ‘Social dysfunction’ (3 items), ‘Anxiety and Depression’ (3 items), ‘Self-Efficacy’ (3 items) and ‘Capable’ (3 items). Structural equation modelling assessed the predictive utility of the factors identified with CFA by using goodness-of-fit criteria (Table 3). The best fitting model was Model 3 with the most acceptable values (Figure 1) and the highest overall CD or *R*-squared as 0.972 (Online Appendix 1, Supplementary files 1).

**TABLE 3:** Goodness-of-fit statistics for tested models of the 12-Item General Health Questionnaire in healthcare workers from South Africa.

Goodness-of-fit statistics	Model 1	Model 2	Model 3
Structural equation model *df*	54	48	48
**Likelihood ratio**			
Chi-squared (LR *X*^2^)	411.41	235.15	222.02
*p*-value	< 0.001	< 0.001	< 0.001
**Population error**			
RMSEA	0.099	0.076	0.074
90% CI, lower bound	0.090	0.067	0.064
Upper bound	0.108	0.086	0.083
*p*-close	< 0.001	< 0.001	< 0.001
**Information criteria**			
AIC	21083.36	20919.09	20905.97
BIC	21245.73	21108.53	21095.45
**Baseline comparison**			
Comparative fit index (CFI)	0.839	0.916	0.921
Tucker–Lewis index (TLI)	0.803	0.884	0.892
**Size of residuals**			
SRMR	0.062	0.048	0.046
Coefficient of determination (CD)	0.856	0.970	0.972

AIC, Akaike’s information criterion; BIC, Bayesian information criterion; SRMR, standardised root mean square residual.

The retained results from the SEM are presented in Model 3 of Figure 1. ‘Social dysfunction’ was positively and statistically significantly associated with ‘lost much sleep over worry’, ‘felt constantly under strain’, ‘felt that you could not overcome your difficulties’ and ‘been able to concentrate on what you are doing’. ‘Anxiety and Depression’ was positively and strongly associated with ‘been feeling unhappy and depressed’, ‘been losing confidence in yourself’ and ‘been thinking of yourself as a worthless person’. ‘Self-Efficacy’ was positively and statistically significantly associated with ‘felt that you are playing a useful part in things’ and ‘felt capable of making decisions about things’. ‘Capable’ was associated and statistically significant with ‘been able to enjoy your normal day-to-day activities’ and ‘been able to face up to your problems’.

## Discussion

This was the first evaluation of the factor structure of the GHQ-12 in a South African population that found good internal consistency for the GHQ-12, reliability and validity of the GHQ-12 among HCWs. The EFA identified the factors and the CFA established the factor structure. The factor structure was not unitary (one factor distress) but a multidimensional scale made up of four factors: Social Dysfunction (Factor I), Anxiety and Depression (Factor II), Self-Efficacy (Factor III) and Capable (Factor IV) with a robust external validity for all four factors identified.

This study demonstrated adequate reliability and validity for a professional South African population and demonstrated the best-fit data with multidimensional structure of four factors similar to a multicentre study involving Ethiopian populations made up of Social Dysfunction, Anxiety and Depression, Self-Efficacy and Capable (Gelaye et al., 2015) and to the literate Kenyan study (Abubakar & Fischer, 2012).

The Cronbach’s alpha and the McDonald Omega coefficient are measures of the composite reliability for internal consistency of the GHQ-12 demonstrated a reliability higher than 0.70 and the two estimators were compared in this study. Similar results were found in another study consisting of a random sample of Brazilian physicians registered in the medical Council system (Oliveira et al., 2023) and a similar Cronbach’s alpha was found in primary care patients from Indonesians (Anjara et al., 2020).

Unlike the unidimensional factor structure reported in an Indonesian population (Anjara et al., 2020), the bi-factor structure in the Brazilian study (Oliveira et al., 2023) and the Norwegian Navy (Hystad & Johnsen, 2020), this study suggested a four-factor structure similar to the multidimensionality reported in the Spanish population (Sánchez-López & Dresch, 2008). The SEM conducted in this study did not include insomnia and mental health problems (HSCL-25) as in unidimensional domains (Hystad & Johnsen, 2020; Skogen et al., 2017) but rather opted for the multidimensionality as obtained from the initial results of the EFA and CFA.

This study showed a four-factor model made up of Social Dysfunction (Factor I), Anxiety and Depression (Factor II), Self-Efficacy (Factor III) and Capable (Factor IV) as the best explanation of a sample of South African HCWs compared to the three-factor model labelled as Anxiety-Depression, Social Dysfunction and Loss of Confidence when testing for the six-factor analytic models (Shevlin & Adamson, 2005) and reported in longitudinal and cross-sectional studies (El-Metwally et al., 2018; Liang et al., 2016; Mäkikangas et al., 2006). This study confirmed the valid use of GHQ-12 in a professional occupational group as previously shown in a study of young civil servants in China, but differed in the suggested factor structure, which was three-factor compared to the four-factor structure in this study (Liang et al., 2016).

A Finnish study using GHQ-12 and GHQ-20 concluded that the GHQ-12 had a three-factor structure and the GHQ-20 had a four-factor structure, which was superior to the GHQ-12 as it provided an additional factor named anhedonia suggesting some discriminative power (Penninkilampi-Kerola et al., 2006). The aforementioned studies that suggested a three-factor structure provided little information beyond that of a general factor while this study showed more information with the four-factor structure after SEM. The three-factor structure was similar to this four-factor structure with similar loadings but with differences regarding load on each factor. The main difference was observed in the factor orderings such that, the 3 items in Factor II (Anxiety and Depression) were the same as 3 of the 4 items in Factor II and 2 items in Factor I (Social Dysfunction) were similar to the 2 of 3 items in Factor III (Sánchez-López & Dresch, 2008).

The data were also explored for modification indices (MI) suggesting covariance between ‘Been losing confidence in yourself?’ and ‘Been thinking of yourself as a worthless person?’ (MI:40.87), ‘Been feeling unhappy and depressed?’ and ‘Been thinking of yourself as a worthless person?’ (MI:26.51), and ‘Felt constantly under strain?’ and ‘Been thinking of yourself as a worthless person?’ (MI:27.87) (Online Appendix 1, Supplementary files 2). This may be explained by the existence of an unspecified factor not included in the model that might partially account for the relationship between the variables or measurement artefacts. The existence of between-factor differences suggests that the GHQ-12 has multidimensional characteristics not captured by a severity score as reported by Vanheule and Bogaerts in a Belgian sample (Vanheule & Bogaerts, 2005) and Graetz in an Australian sample of young people (Graetz, 1991).

The GHQ-12 is a relatively brief tool, can easily be scored and has been previously used in different settings for screening purposes to detect psychological distress. The adequacy of the GHQ-12 was first tested and the assumptions for inferential analysis in South African HCWs were assessed. The EFA identified the factor structure and the CFA tested the theoretical foundation of the hypothesised factor structure further establishing the model fit.

### Limitations of the study

The interpretation of the factors is complicated by the lack of prior knowledge leading to difficulty in interpretation of the results obtained from EFA. However, the use of CFA and SEM with standardised coefficients permitted the confirmation of the factors. The longer versions of the GHQ are useful in assessing the degree of psychological morbidity and outcomes for clients managed at mental services (Campbell et al., 2003). The primordial strength of this study is the validation of the utility of the GHQ-12 among South African HCWs. The limitation of this study includes the non-generalisability of the results to the whole South African and African population for screening as this study was limited to HCWs only. There may also be differences in the various professional disciplines such that what nurses may experience might differ from the experiences of medical doctors because of the nature of their roles.

## Conclusion

The GHQ-12 displayed adequate reliability and validity in measuring psychological distress among South African HCWs. The factor structure suggested multidimensionality rather than a unidimensional construct. The findings of this study affirm the effectiveness of the GHQ-12 in a professional group of South Africans. The GHQ-12 can be a useful screening instrument in the South African population for general symptoms of mental distress to effectively assess overall psychological well-being and detect non-psychiatric challenges. Further research is warranted with larger sample sizes to test the reliability and validity of the GHQ-12 in the general South African population.
